# Examining the mechanisms by which women’s status and empowerment affect skilled birth attendant use in Senegal: a structural equation modeling approach

**DOI:** 10.1186/s12884-017-1499-x

**Published:** 2017-11-08

**Authors:** Kyoko Shimamoto, Jessica D. Gipson

**Affiliations:** Fielding School of Public Health, University of California, Los Angeles, 650 Charles E. Young Dr. South, 16-035 Center for Health Sciences, Los Angeles, CA 90095-1772 USA

**Keywords:** Maternal mortality, Delivery care, Skilled birth attendant, Women’s status, Women’s empowerment, Structural equation modeling, Sub-Saharan Africa

## Abstract

**Background:**

Despite the reduction in maternal deaths globally, maternal mortality rates remain unacceptably high, particularly in some regions of the world. In sub-Saharan Africa, maternal mortality rates have even increased recently, with 201,000 deaths in 2015 as compared to 179,000 in 2013. Use of a skilled birth attendant (SBA) at delivery has remained low, despite evidence of the effectiveness of SBAs in reducing maternal deaths. Women’s empowerment is increasingly recognized as a key determinant of maternal health care-seeking and outcomes, yet empirical examinations of the linkages between women’s empowerment and delivery care use are particularly limited, especially from sub-Saharan Africa.

**Methods:**

Using data from the 2010 Senegal Demographic and Health Survey (*n* = 7451), in this study we employed structural equation modeling (SEM) to investigate the complex and multidimensional pathways by which three women’s empowerment domains (household decision-making, attitudes towards violence, and sex negotiation) directly and indirectly affect SBA use.

**Results:**

Although variations were observed across measures, many of the women’s status and empowerment measures were positively related to SBA use. Notably, women’s education demonstrated a substantial indirect effect: higher education was related to older age at first marriage, which was associated with higher levels of empowerment and SBA use. In addition to age at first marriage, gender-role attitudes (e.g., progressive attitudes towards violence and sex negotiation) were significant mediators in the relationship between education and SBA use. However, household decision-making was not significantly associated with SBA use.

**Conclusions:**

Findings indicate significant effects of women’s education, early marriage, and some dimensions of women’s empowerment on SBA use. SEM was particularly useful in examining the complex and multidimensional constructs of women’s empowerments and their effects. This study informs policy recommendations and programmatic efforts to reduce maternal mortality in sub-Saharan Africa by strengthening support for women’s access to higher education, delaying marriage and childbearing among girls and young women, and supporting more equitable gender norms.

**Electronic supplementary material:**

The online version of this article (doi:10.1186/s12884-017-1499-x) contains supplementary material, which is available to authorized users.

## Background

Maternal mortality remains unacceptably high in low- and middle-income countries, where 99% of maternal deaths occur. In 2015, 303,000 women died from causes related to pregnancy and childbirth globally, most of which were preventable. Sub-Saharan Africa (SSA) has some of the highest rates of maternal mortality, with an average maternal mortality ratio (MMR) of 546 (per 100,000 live births), as compared to the global average of 216. The risk of maternal death has even increased most recently in SSA, and stark disparities remain in the lifetime risk of maternal death when comparing SSA (1 in 36) to the global average (1 in 180) [1].

Delivery assistance by a skilled health professional is critical to reduce maternal and newborn deaths: it is the most effective and cost-effective approach [2–4]. The global World Health Organization (WHO) definition of a skilled birth attendant (SBA) is “an accredited health professional — such as a midwife, doctor, or nurse — who is proficient in the skills needed to manage normal (uncomplicated) pregnancies and childbirth, and to identify, manage and refer complications in women and newborns” [2]. Availability and quality of delivery care with an SBA is estimated to avert 16–33% of maternal deaths [5].

Despite such evidence, levels of SBA use remain low, particularly in SSA where only half of deliveries have an SBA present [6]. In SSA and other low-resource settings, further reductions in maternal mortality are inhibited by a complex set of factors that affect women’s access to and use of maternity health care services. For example, low socioeconomic status (low education and economic status), long distances to facilities, insufficient transportation, lack of qualified staff and supplies, and poor quality of care are negatively associated with delivery care use and health outcomes for mothers and newborns [7–10]. There is growing evidence that women’s social status and the power they wield in their households, communities, and societies are key determinants of delivery care use and its associated health outcomes [7, 10–13], which are necessary to achieve social development, economic growth, and poverty reduction [14].

Women’s status and empowerment are inherently complex constructs referred to in the empirical literature that serve to represent the social standing of women in society and their ability to participate in decision-making and to take action on issues affecting their own wellbeing and that of their families. *Women’s status* is generally understood as “women’s overall position in society” [15] and is often operationalized as women’s education, economic status, or employment [16]. On the other hand, *women’s empowerment* is defined as “women’s ability to make strategic life choices” — comprising three interrelated dimensions such as resources, agency, and achievements [16]. The existing literature often uses proxy measures for “resources” and “agency,” including women’s participation in household decision-making, access to and control over household resources, and attitudes towards gender relationships [11, 16, 17].

Despite the recognized importance of women’s status and empowerment on health and social outcomes, the empirical literature examining the impacts of women’s empowerment is insufficient in some substantive areas, and specific geographic regions are underrepresented in the literature. Although increasing, there are relatively few studies on the relationships between women’s empowerment and delivery care use (i.e., presence of an SBA at delivery, institutional delivery) from African contexts despite increasing programmatic efforts and evidence indicating the influence of gender norm transformation on reproductive, maternal, and child health in SSA and elsewhere [18, 19]. In the subset of SSA studies that examine women’s empowerment as a multidimensional construct, we find unique and disparate effects of empowerment dimensions on delivery care use [4, 20–23]. For example, a multi-country study in eight African countries found varied influences of women’s empowerment domains (i.e., household decision-making, financial decision-making, attitudes towards violence and sex negotiation) on the likelihood of women delivering at a health care facility [20, 22]. In particular, women’s household decision-making was positively associated with delivery at a health facility only in Nigeria, yet in the rest of the seven countries this showed no significant effect on facility-based delivery care.

Examination of the linkages between women’s empowerment and SBA use is particularly important in Senegal, where continuous social and development efforts towards gender equality are resulting in gradual shifts in gender norms and relations [24–26]. Women’s higher social status has been found to be a critical determinant of facility-based delivery care-seeking in Senegal [27, 28], suggesting that efforts to improve gender equality may also further promote SBA use.

The varied effects of women’s empowerment on maternal health care-seeking may be due, at least in part, to two key methodological challenges in this area of study. First, there are inconsistent operationalizations and measurements of empowerment across studies and settings [11, 17]. Despite the importance of capturing the multiple dimensions of empowerment [16], summative measures or composite indices are often used that likely mask the unique and potentially countervailing influences of each empowerment dimension, though there are some exceptions of studies that have incorporated multidimensional measures [4, 20–23, 29, 30].

Second, although structural equation modeling (SEM) has been used to examine mechanisms influencing pregnancy and child health in studies from higher income countries [31, 32], there are no known studies using SEM that empirically test the complex linkages between multidimensional measures of empowerment and delivery care use from low-income countries.

Given these identified gaps in the literature, this study employed SEM and aimed to examine the mechanism linking women’s status, empowerment, and SBA use in Senegal, a setting where women’s status and empowerment remain constrained and a substantive proportion of births are unattended by an SBA. In particular, this study first examined the relationship of women’s education, age at first marriage, and multiple empowerment dimensions with SBA use. Second, the mediation effects of age at first marriage and empowerment dimensions were assessed as potential intervening constructs in the pathway between women’s education and SBA use.

## Methods

### Study setting and data

Although maternal mortality has declined in Senegal over the last two decades (from 540 in 1990 to 315 in 2015) [1], rates still exceed those in other regions. Further reductions in maternal mortality likely hinge on increasing SBA use at delivery. More than one-third of women do not have a delivery attended by an SBA, and there are stark disparities in access to care by sociodemographic characteristics, including geographic residence (e.g., urban-rural) and household wealth [26].

This study used data from the 2010–2011 Senegal Demographics and Health Survey (DHS), a nationally representative household survey [26]. The study sample comprises currently married women who had at least one birth during the 5 years preceding the survey. Unmarried women were dropped from the analysis, because questions on household decision-making were asked of married women only. Also, women with missing data on the decision-making, gender attitudes, and sociodemographic variables were dropped, yielding a final analytic sample of 7451. The proportion of missing observations is marginal — only 1.6 percent — thus, the potential bias due to missing data is negligible.

### Conceptual framework

**Fig. 1:**
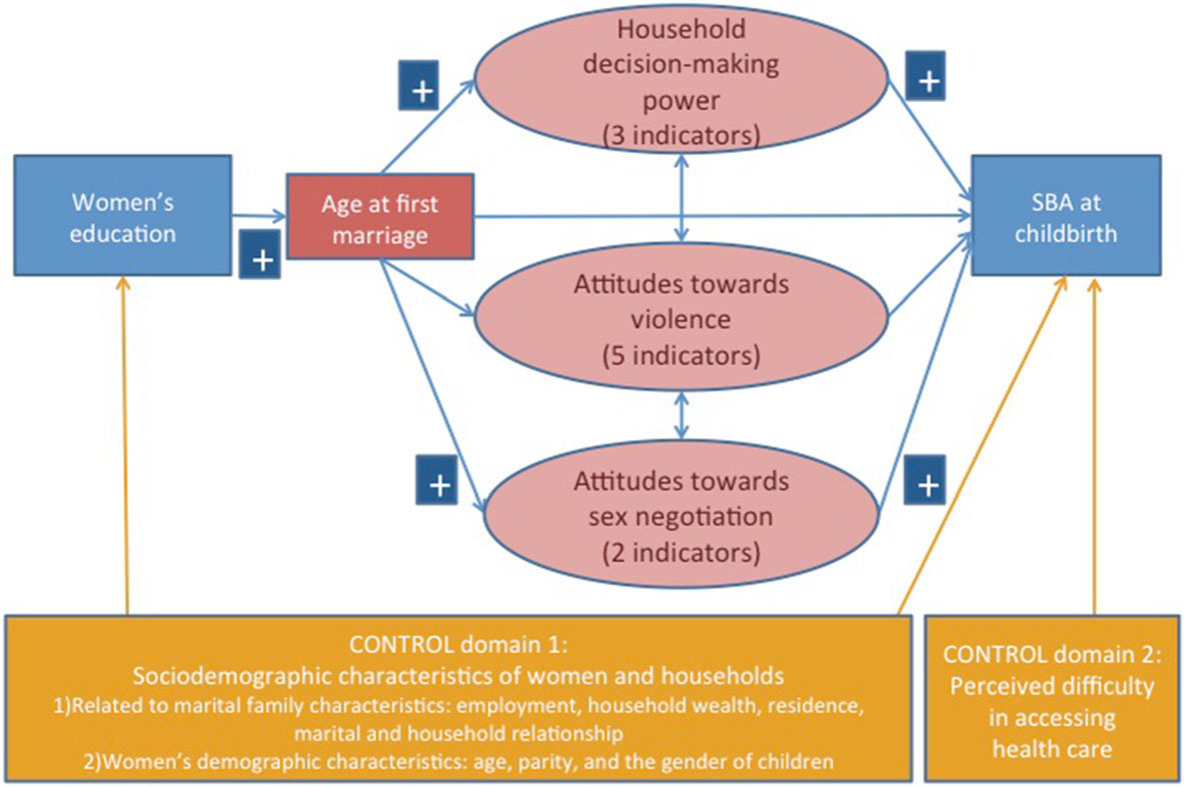
A conceptual framework on women’s status, empowerment, and SBA use

This analysis employed an integrated conceptual framework to examine the pathways linking women’s status and empowerment to SBA use, based on the existing conceptual definitions and the Theory of Gender Stratification [16, 33]. As shown in Fig. 1, women’s status, operationalized as women’s education, positively affects multiple dimensions of empowerment, which in turn promotes SBA use. According to the theory, women with greater power have more control over their lives and a variety of “life options” [33, 34]. Household decision-making power and attitudes towards gender norms represent “agency” and “resource” dimensions of empowerment, and they are frequently examined in studies using DHS data. This framework also considers the potential negative influence of early marriage on empowerment and delivery care use. The potential confounding effects of sociodemographic characteristics of women and households were controlled, as well as perceived difficulty in accessing health care.

### Analytic strategy and measures

A latent variable SEM was employed for this study, which included both a structural portion (i.e., with measured variables) and a measurement portion (i.e., with latent constructs). The latent variable SEM comprised three latent constructs, each of which included individual empowerment indicators to represent each empowerment dimension. The latent variable SEM included five “endogenous variables” (those that appear as dependent variables in at least one equation in the SEM) and multiple “exogenous variables” (those that are never dependent variables in the SEM) [35]. Endogenous variables were SBA use, household decision-making, gender-role attitudes, and age at first marriage. Exogenous variables were women’s education, sociodemographic characteristics of women and households, and perceived difficulty in accessing health care. Operationalizations of these measures were determined based on the existing theory and literature review and have been employed in a separate analysis by the authors [4].

#### Endogenous variables

*SBA use* at childbirth was operationalized as the use of SBA(s) at the last childbirth per the WHO definition [2]. SBAs included doctors or assistant medical officers, clinical officers, nurses, or midwifes; non-SBAs included village health workers, traditional birth attendants, relatives or friends, others, or no one at the delivery [36]. Based on these answer options in the survey, the variable was recoded into binary form.

*Age at first marriage* was examined as a continuous variable. In the survey, MEASURE DHS calculated the age at first marriage based on the date of the first marriage or union (“living with a man as if married”) and the respondent’s date of birth [36].

*Household decision-making power* was examined as a latent construct consisting of three indicators: women’s participation in decisions regarding their own health care, major household purchases, and visits to family/relatives [36]. These questions were asked only to women who were currently married/in union. The variables were first recoded to examine if women participated in decisions (i.e., alone/jointly with their husband) or not. A latent variable was constructed from the three binary variables for each decision.

*Attitudes towards violence* were examined as a latent construct consisting of five indicators concerning a woman’s acceptance of wife-beating by her husband/partner. The survey asked about the following five situations: if she goes out without telling him, neglects the children, refuses to have sex with him, argues with him, or burns the food [36]. Each variable was first recoded into binary data (i.e., yes accept, or no).

*Attitudes towards sex negotiation* were also assessed as a latent construct based on two questions. The questions assessed a woman’s perceived ability to negotiate sexual relations, and the following two aspects were asked: if the respondent can refuse having sex or can ask her husband to use a condom [4, 36]. Each variable was first recoded into binary form: the respondent can refuse/ask, or not (which included responses as cannot refuse/ask, don’t know, not sure, or depends).

#### Exogenous variables: women’s education and control variables

*Women’s education* was measured as a continuous variable representing the number of years that the woman attended school. *Sociodemographic characteristics of women and households* were included in the model as control variables, as also employed in the separate analysis by the authors [4]: *women’s age, parity, employment for payment, household wealth, marital and household relationship, gender composition of children, place of residence, and a woman’s education relative to her husband. Women’s age* and *parity* (i.e., the birth order of the child) were examined as continuous variables due to linear relationships with SBA use identified in descriptive analyses. *Employment for payment* was a binary measure that examined if a woman had been employed (for cash or in-kind), or not, in the last 12 months. *Household wealth* was determined based on reported ownership of household assets (e.g., consumer items and home attributes). A composite index was created by MEASURE DHS, based on the results of principal component analysis and divided into quintiles [36]. *Marital relationship* was included as the following: monogamous union, polygamous as a first wife, or polygamous as a second wife or lower, to consider differences by the type of relationship and wife order given evidence of differences in women’s status and power across these relationship types [37–39]. *Household relationship*, a binary variable, was examined if the respondent was a household head or not. The *gender composition of children* examined if the respondent had at least one living son or not, because having a son has been shown to be an important reflection of African women’s status and power [40]. The *place of residence* was a binary measure; it denoted if the respondent lived in an urban or rural area. *Woman’s education relative to husband’s* was assessed as continuous, whereas relative age was assessed but not included in the final model due to non-significant results.

*Perceived difficulty in accessing health care* was also included as a control variable, and the survey examined the following four aspects: getting permission to go; distance to the health care facility; getting money needed for advice/treatment; or not wanting to go alone [36]. The first item on permission was somewhat related to decision-making as a construct of empowerment, while the other three items measured broader socioeconomic factors particularly related to financial and geographic access to health facilities. Each variable was first recoded into a binary variable to demonstrate if the respondent perceived a big problem, or not (i.e., including the answer options of “not a big problem” and “not a problem at all”). A continuous variable showed the number of aspects for which the respondent perceived difficulties (scored 0–4).

### Analytic models and steps

Data analysis consisted of three steps. The first step was to undertake descriptive analyses using SAS 9.3. The second step was to conduct factor analyses using Mplus version 7.3 with a geomin rotation. Exploratory factor analysis (EFA) identified the number of factors/latent constructs and the underlying factor structure of empowerment, and then three-factor confirmatory factor analysis (CFA) examined the appropriateness and generalizability of the measurement portion of the SEM. The third step was to employ SEM with Mplus to examine the pathways from women’s education to SBA use. All the analyses were conducted using polychoric correlations for categorical variables, and accounted for individual weights and a two-stage cluster sampling design to address disproportionate sampling and design effects of more than 1.00 [41].

The SEM separately regressed SBA use, household decision-making power, attitudes towards violence, attitudes towards sex negotiation, and age at first marriage using probit regression with weighted least squares means and variance adjusted (WLSMV). This SEM employed listwise deletion with missing observations. In the model, all of the exogenous variables were designated as covarying because of potential relatedness. Also, the errors/disturbances of empowerment dimensions were all covarying, as the unobserved aspects of these constructs were also likely to be interrelated.

Model fit was assessed using recommended model fit indices: root mean square error of approximation (RMSEA) < 0.06 (or less as a “close” fit) and a comparative fit index (CFI)/Tucker-Lewis index (TLI) ≥ 0.95 [42]. Weighted root mean square residual (WRMR) (less than 0.90) was also calculated by Mplus for the models with categorical endogenous variables [43].

## Results

### Descriptive results

Around two-thirds of the respondents used an SBA at the last birth (Table 1). Overall, women’s reported levels of household decision-making were low, and gender norm attitudes were permissive. Women got married or started a union at mean age 18.3 years and had on average less than 2 years of formal education.

**Table 1:** Characteristics of participating, currently married women with at least one birth in last 5 years (*n* = 7451), Senegal DHS 2010

Variables	Freq.	Weighted	SE
		Mean or proportion	
Outcome			
Skilled birth attendant use at the last childbirth	4251	66.30	1.27
Mediators: women's empowerment measures			
Household decision-making (mean, scored 0–3)		0.92	0.03
Attitudes towards violence (mean, 0–5)		2.80	0.05
Attitudes towards sex negotiation (mean, 0–2)		0.60	0.02
Age at first marriage (mean)		18.29	0.10
Demographics and perceived accessibility of health care			
Education (mean in years)		1.79	0.08
Current age		29.40	0.12
Household wealth quintile			
Poorest	2264	22.38	1.31
Poorer	1882	20.95	1.18
Middle	1534	19.19	1.13
Richer	1056	19.85	1.34
Richest	715	17.63	1.12
Employment for payment			
Currently employed or employed last 12 months	3386	46.04	1.12
Parity (total number of children ever born)		3.81	0.04
Marital relationships			
Monogamous union	4909	68.19	0.83
Polygamous as 1st wife	991	12.73	0.44
Polygamous as 2nd or lower	1550	19.08	0.55
Household head	322	4.98	0.38
Place of residence			
Urban	2267	39.95	1.62
Rural	5184	60.05	1.62
Perceived difficulty in accessing health care (Mean, scored 0-4)		1.23	0.04

Frequency missing = 119 (with attitudes towards violence), 1 (with marital relationships)

*SE* standard error

### Factor analysis results

The EFA results indicated three factors (eigenvalues > 1.0) [44]: (1) *household decision-making power* (three indicators), (2) *attitudes towards violence* (five indicators), and (3) *attitudes towards sex negotiation* (two indicators). See Table 2. The three factors were significantly, yet not highly, correlated with factor correlations less than 0.31 (*p* < 0.05), indicating the distinction of each factor.

**Table 2:** Factor analysis for indicators of empowerment (*n* = 7451), Senegal DHS 2010–2011

Latent construct	Aspects asked about by survey	Factor loadings (EFA)	*t* value (CFA)
Household decision-making	Decision on own health care	0.916	–
	Decision on major household purchases	0.869	38.927
	Decision on visits to family or relatives	0.851	42.613
Attitudes towards violence	Violence if she goes out without telling her husband	0.917	–
	Violence if she neglects the children	0.933	101.880
	Violence if she argues with him	0.963	122.107
	Violence if she refuses to have sex with him	0.911	101.322
	Violence if she burns the food	0.822	63.985
Gender norms for sex negotiation	Perceived ability in refusing sex	0.803	–
	Perceived ability in asking for condom use	0.771	8.305

In the CFA, the path of the first indicator is constrained to 1 (thus *t* value was not calculated). Significance of *t* values refers to unstandardized parameter values. All the factor loadings are significant at *p* < 0.05. RMSEA = 0.016, CFI = 0.998, TLI = 0.997, WRMR = 1.012

### SEM results

**Fig. 2:**
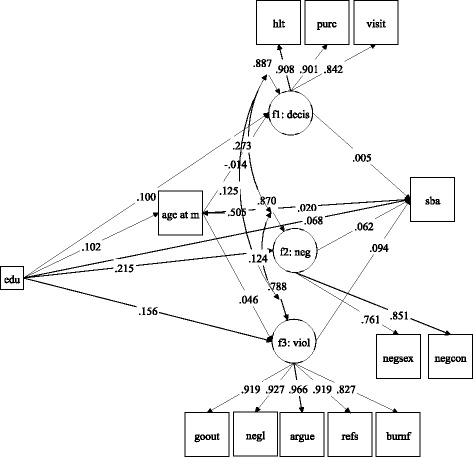
A diagram of the latent variable SEM. *sba* SBA use, *f1:decis* decision-making power, *f2:neg* attitudes towards sex negotiation, *f3:viol* attitudes towards violence, *age at m* age at first marriage, *edu* education. Standardized path coefficients are reported with the following significance levels: ***p < 0.001, **p < 0.01, *p < 0.05. Factor loadings and correlations among disturbances are all significant at *p* < 0.001. Control variables are not included in the figure

The final adjusted SEM results are presented in Table 3 and Fig. 2. The standardized path coefficients and *p* values in the unstandardized metric are reported. The model fit statistics (RMSEA, CFI/TLI, WRMR) show that the models fit the data well.

**Table 3:** Standardized path coefficients of the latent variable SEM (*n* = 7451), Senegal DHS 2010–2011

Predictors in the equation (*X*):	Dependent variables in the equation (*Y*):				
	[Column 1] Age at first marriage	[Column 2] Decision-making power	[Column 3] Attitudes towards violence	[Column 4] Attitudes towards sex negotiation	[Column 5] SBA use
Endogenous variables					
(1) Age at first marriage		–0.014	0.003	0.046*	0.020
(2) Decision-making power					0.005
(3) Attitudes against violence					0.062*
(4) Attitudes towards sex negotiation					0.094***
Exogenous variables					
Education	0.102***	0.100**	0.215**	0.156***	0.068*
Age	0.877***	0.097*	0.006	0.026	0.103**
Parity	–0.749***	0.042	–0.031	–0.020	–0.122***
Employment for payment	–0.022	0.160***	0.028	–0.016	–0.039*
Household head	–0.025*	0.070***	–0.018	0.011	0.005
Urban residence	0.008	0.101*	0.092*	0.093**	0.170***
Having son(s)	–0.020	–0.002	–0.022	0.010	–0.047**
Household wealth (the 2nd lowest)	0.026*	–0.063*	0.001	0.086***	0.150***
Household wealth (the 3rd lowest)	0.047***	–0.034	–0.021	0.117***	0.261***
Household wealth (the 4th lowest)	0.066***	–0.013	–0.005	0.233***	0.342***
Household wealth (the highest)	0.047*	0.040	0.074	0.260***	0.407***
Polygamous union as a first wife	–0.090***	–0.012	0.005	–0.024	–0.039*
Polygamous union as a second or lower	–0.027*	–0.021	–0.034	–0.070***	–0.045**
Less education than husband	0.042*	0.018	0.103***	0.048**	–0.004
More education than husband	–0.014	–0.009	–0.018	–0.008	–0.070**
Perceived difficulty in accessing health care					–0.067***

***p < 0.001, **p < 0.01, *p < 0.05. Reference groups: residence = rural; household wealth = the lowest; marital relationship = monogamous union; relative education = almost the same education as husband

DF (Degree of Freedom) = 109, RMSEA = 0.011, CFI = 0.994, TLI = 0.991, WRMR = 0.915

*R* squared: 0.495 (age at first marriage), 0.113 (decision-making), 0.212 (attitudes towards violence), 0.130 (attitudes towards sex negotiation), 0.457 (SBA use)

Women’s education and gender-role attitudes were positively related to SBA use (Column 5 in Table 3). However, neither age at first marriage nor decision-making were directly related to SBA use. In addition, several sociodemographic variables were significantly related to SBA use, including women’s age, urban residence, and higher household wealth. On the other hand, parity, paid employment, having living son(s), being in a polygamous union, husbands’ lower education relative to women’s, and perceiving difficulties in accessing health care were negatively related to SBA use.

Also, education and several other variables were positively related to older age at first marriage (Column 1 in Table 3). Significant and positive covariates in predicting age at first marriage included women’s older age, higher wealth, and husband’s higher education relative to women, whereas negative covariates were parity, heading household, and being in a polygamous union. Further, education was positively associated with all three empowerment dimensions: decision-making, attitudes towards violence, and attitudes towards sex negotiation (Columns 2–4, Table 3).

Moreover, apart from education, other sociodemographic variables were significantly, yet differently, related to the empowerment dimensions. Significant covariates in predicting decision-making included older age, paid employment and urban residence. Progressive attitudes towards violence were related to older age at first marriage, urban residence, higher wealth, and husband’s higher education relative to wife, whereas polygamous marital relationship as second or lower wife order was associated with permissive attitudes. Concerning attitudes towards sex negotiation, urban residence and husband’s higher education were also significantly and positively associated (Columns 2–4). Besides the significant correlations among the three empowerment dimensions per the factor analysis result, the SEM analysis showed that the residuals of these three factors were also significantly correlated with each other.

The direct and indirect effects of women’s education on SBA use from the latent variable SEM are presented in Table 4. In addition to its direct effect, the total indirect effects of education on SBA use through all the identified pathways were significant. In particular, age at first marriage and attitudes towards violence and sex negotiation significantly mediated the relationship between education and SBA use. For example, women’s higher education was related to older age at first marriage, which in turn positively influenced progressive attitudes towards violence and SBA use. The proportion of the indirect effect of education over the total effect was substantial (31.3%).

**Table 4:** Summary of standardized direct and indirect effects of education on SBA use (*n* = 7451), Senegal DHS 2010–2011

		Coefficient	*t* value
Total effect		0.099	2.989***
Direct effect		0.068	2.006*
Total indirect effect		0.031	4.582***
Indirect effect via:	Age at first marriage	0.002	1.003
	Decision-making power	0.000	0.166
	Attitudes towards violence	0.015	3.217***
	Attitudes towards sex negotiation	0.013	2.242*
	Age at first marriage then decision-making power	0.000	–0.161
	Age at first marriage then attitudes towards violence	0.000	1.975*
	Age at first marriage then attitudes towards sex negotiation	0.000	0.082

***p < 0.001, **p < 0.01, *p < 0.05

## Discussion

This study examined the pathways linking women’s status and empowerment to SBA use. The analysis provided evidence of the direct and indirect effects of education on SBA use through multiple empowerment dimensions, demonstrating evidence of potential causal mechanisms affecting SBA use. SEM is uniquely equipped to examine such complex mechanisms, by identifying and measuring intervening effects which are rarely empirically tested.

Four key findings arise from this analysis. First, the study showed the significant and positive effect of women’s education on SBA use, with a significant proportion of indirect effects of education operating through the empowerment dimensions. This finding is generally consistent with previous evidence that women’s education, as well as other sociodemographic characteristics, affect delivery care use and outcomes [4, 8, 20–23, 30, 45–50]. Evidence from this study suggests the influence of education as a key, direct driver for increasing delivery care use from skilled providers, possibly by advancing knowledge generally on the importance of seeking care for delivery care services as a means of mitigating risks of potentially life-threatening complications for mother and baby. Further, the indirect effects of education on delivery care use through age at first marriage and gender-role attitudes (towards violence and sex negotiation) underscore the importance of policy and program interventions to promote women’s education for improving women’s health, through women’s empowerment and gender equity [6, 51–53].

Second, the significant influence of age at first marriage on delivery care use — directly and indirectly — is in alignment with increasing evidence of the critical influence of early marriage and childbearing on empowerment, delivery care use, and other reproductive health behaviors and outcomes [51, 54]. This study highlights the important linkages such that older age at first marriage is related to progressive gender-role attitudes, leading to higher likelihood of SBA use and likely better delivery outcomes.

Third, the evidence from this study highlights the critical role of gender-role attitudes in promoting delivery care-seeking in Senegal, and possibly other settings, where permissive gender norms persist. This finding is consistent with successes from sexual and reproductive health programs (e.g., HIV/AIDS prevention) which focused on transforming gender norms and integrating men as supportive partners [19, 55, 56], as well as recent evidence from SSA finding positive influences of progressive gender-role attitudes on delivery care use [4, 20–22].

Fourth, the use of SEM in this analysis provided the simultaneous comparison of several empowerment dimensions on SBA use, clearly outlining variations in the magnitude and significance of each domain. The variations in the effects of independent empowerment domains on SBA use affirm the importance of operationalizing and measuring women’s status and empowerment as a multidimensional construct, and the utility in identifying specific constructs or areas for subsequent intervention and policy efforts. For example, findings from this study suggest a prioritization of programs focusing on gender norm transformation and gender equality in Senegal, especially to promote equitable sexual negotiations between couples.

The null effects of household decision-making on SBA use, however, were unexpected yet consistent with previous studies showing an inconsistent influence of decision-making on delivery care use [4, 20–22]. Our analysis using hierarchical multivariate regression also showed no effect of decision-making on SBA use in a model excluding the two attitudinal measures of empowerment, while there was a significant bivariate relationship [4]. As noted by other scholars, the decisions on health care-seeking, particularly in a resource-constrained setting, are complex and contingent upon multiple logistical and structural factors [7, 10, 13]. Thus, the null findings from this analysis may be due to the limitation of existing measures in the survey. Indeed significant correlations among the residuals for these three empowerment dimensions suggest that there may be similar or shared features in the unexplained aspects of these factors. Further empirical and programmatic investigations are essential given increasing recognition of the role of women’s status and empowerment as a means of achieving health and broader development goals [13, 14, 52, 53].

This study entails some limitations. First, despite the rigor of SEM, any causal inference is tentative, and potential reciprocal effects may exist, especially given the cross-sectional nature of the DHS survey data. However, relevant theories and descriptive results support the hypothesized causal relationships. Second, the operationalization and measurement of women’s status and empowerment was limited by the available measures from the DHS, of which the relevance has been generally supported in Asia but less so in Africa [57]. Multi-dimensionality of empowerment has been underscored by the conceptual definition by Kabeer (2001) (e.g., “agency”, “resources”, “achievement”) [16, 33], which could have been better captured in the survey. Third, the representativeness of the study sample and generalizability of the results are limited due to the omission of currently unmarried women. These women contributed 7.1% of all births that occurred (*n* = 576) in the study, yet since unmarried women were excluded from the decision-making questions, they were excluded from the analytic sample. Perhaps even more important to address in future program and research efforts is the omission of adolescents aged 10–14. Young adolescents are not interviewed in the DHS and other global surveys, despite the fact that a proportion of young adolescents will enter into marital relationships and begin childbearing at these ages in Senegal and several other African contexts. The underrepresentation of adolescents is critical, especially in light of growing evidence that adolescents are at greater risk of delivery without skilled professionals, unsafe abortion, and maternal deaths [51, 58–61].

## Conclusions

Despite these weaknesses, this study is one of the first to use nationally representative data to examine complex pathways between women’s empowerment and SBA use using SEM. The findings confirm the multidimensionality of empowerment and the need to examine critical intervening factors leading to reproductive and maternal health service use and outcomes. Moreover, this study highlights the need for policy and program interventions to support women’s access to higher education, to delay marriage and childbearing among girls and young women, and to transform gender norms as a means of increasing SBA use and accelerating reductions in maternal mortality in Africa.

## Additional file

Additional file 1: Open peer review. (PDF 155 kb)

## Abbreviations

DHS: Demographic and Health Survey(s)
SBA: Skilled birth attendant
SSA: Sub-Saharan Africa
