# Supplementary material for: Examining the mechanisms by which women’s status and empowerment affect skilled birth attendant use in Senegal: a structural equation modeling approach
Source: BMC Pregnancy Childbirth. 2017 Nov 8;17(Suppl 2):341. doi: 10.1186/s12884-017-1499-x (PMC5688451; doi:10.1186/s12884-017-1499-x)
Supplement: Additional file 1: — Open peer review. (PDF 155 kb) [file 12884_2017_1499_MOESM1_ESM.pdf]

Reviewer reports

Title: Examining the mechanisms by which women's status and empowerment affect skilled birth attendant use in Senegal: a Structural Equation Modeling approach

Reviewer 1: Saifuddin Ahmed

This is a well-crafted manuscript. The study used appropriate statistical methods and adequately reported the SEM model fit indices. I have the following suggestions to improve the manuscript:

1. The study is based on a country (Senegal) and may be inappropriate to consider that the study is on West Africa. Either refer the study for "…a country in West Africa" or "in Senegal" in the title.
2. A major omission of the paper is a conceptual framework, which may serve as the basis of specifying the path diagram. Without a conceptual framework, it is difficult to follow the construct of empowerment, rationales for specifying causal pathways, and justifying the variables included in the SEM. Discussion on the conceptualization, operationalization and the measurement of women's status and empowerment is also scanty in the paper. The authors clearly recognize the problems of "inconsistent operationalizations and measurements of empowerment" (p. 6, line # 83) in the gender literature. A conceptual framework will fill those voids.
3. I do not consider that the inference, "Overall, women's status and empowerment were positively related to SBA use," is appropriate when the result shows decision-making, which is the key component in defining women's status based on actual experience, was not related to SBA use. Among the three construct variables of status and empowerment, the other two are based on perceived attitudes. The interconnections or correlations among the three latent variables are not clear from the presented results. If the two attitudinal latent variables are dropped, does the relationship between decision-making and SBA become significant? Understanding the relationships among these three constructs is critical and relevant for policy implications of this study in advancing women empowerment and gender equity.
4. The study did not explore the linkages between the age at marriage and gender role attitude. Nevertheless, the Discussion section suggested, "This study highlights the important linkages such that older age at first marriage is related to progressive gender-role attitudes, leading to higher likelihood of SBA use and likely better delivery outcomes." I think this beyond the scope of the study results.

Minor suggestions:

1. Label Figure 1 with variable label, not variable name.

2. P.5-6, states, "Examination of the linkages between women's empowerment and SBA use is particularly important in Senegal, where continuous social and development efforts towards gender equality are resulting in gradual shifts in gender norms and relations. For example, women's higher social status is a critical determinant for maternal health care seeking [27, 69 28]." The logical connection between the first sentence and the linking following sentence is not clear to me. Consider the "for example" sentence that is linked to Senegal's context.

3. May consider changing the text,

“All the analyses were conducted accounting for individual weights, clusters (i.e., Primary Sampling Unit), and sample strata using the survey analysis commands” (p.13) to:

“All the analyses were conducted accounting for individual weights and multi-stage stratified cluster survey design for addressing disproportionate (urban-rural) sampling and design-effects >1.00 in the DHS data”

The use of text “using the survey analysis commands” sound like using the software as a black box.

4. Specifying p <. 01 is sufficient for portraying a meaningful statistical significance level (less than 1%). No need to specify *** p <0.001.

I declare that I have no competing interests.

Reviewer 2: Sheri Weiser

This is well written manuscript that investigates associations between several domains of women's empowerment and Skilled Birth Attendant (SBA) use in Senegal. Overall, the methods and analysis are sound, the writing is clear, and the findings have potential policy implications. In terms of approach, strengths include the use of a large population-based representative sample, the low rate of missing data, the use of multiple empowerment domains, and the use of SEM to evaluate potential causal pathways. However, there are several aspects of the manuscript which should be strengthened.

- Major Compulsory Revisions

1) The introduction, though well written, can be condensed and tightened. For instance, they state that the literature linking empowerment on health is "remarkably scant", but they already cited several sources in the previous paragraph (beginning with line 54) on associations between women's social status and health outcomes, and then spend the next few paragraphs talking about all of the various studies linking empowerment indicators and care delivery use both in Senegal and elsewhere in SSA. They can better organize the introduction to more succinctly summarize the existing body of literature on empowerment measures and delivery care use (which should not be referred to as 'scant') before moving to the methodologic limitations with the previous studies that helped to justify the need for the current study.

2) While authors acknowledge the limitation of dropping unmarried women in the limitations section, this is an important limitation that needs to be better addressed. To contextualize this limitation and how it affects the generalizability of their findings, it would be useful to know the proportion of births that occur within and outside of the context of marriage in Senegal.

3) The authors could more clearly lay out their hypothesized conceptual framework in the introduction or methods, along with supporting literature.

4) In methods, "getting permission to go" to the healthcare provider seems like a measure of household decision-making, rather than a control measure of perceived difficulty accessing health care. The authors should better justify the decision to categorize things as they did.

5) In the discussion starting at line 289, the authors discuss how the use of SEM "provided the simultaneous comparison of several empowerment dimensions on SBA use, clearly outlining variations in the magnitude and significance of each domain." Yet, the authors do not elaborate at all on the implications of the variations in the magnitude and significance of each domain for policy and programs.

I also recommend the following minor revisions.

1. The authors often alternate between using ‘Africa’ and ‘Sub-Saharan Africa’ (SSA) (for example, final sentence in first paragraph of background). I recommend that they are consistent, use ‘Sub-Saharan Africa’ throughout.
2. The last sentence in background (lines 101-102) is awkward and could be stated more clearly.
3. I recommend careful editing throughout. A few examples (which are by no means a comprehensive list) include:
   a. In the fifth paragraph of the background, first sentence: “women” is repeated twice (line 60).
   b. In the last sentence in that paragraph (line 65), punctuation needs to be fixed at the end.
   c. Seventh paragraph of the background starting with line 73), the authors should rewrite with consistent use of tenses.
   d. Results section, descriptive results, final sentence (lines 214-215). Writing is awkward. It may read better to say “…and had on average less than two years of formal education.”
4. The labeling of the latent variable SEM analysis (figure 1) could be improved.
5. Line 225---there is a reference to figure 2, which I assume is meant to be for figure 1.

- Discretionary Revisions

1. In the Background, starting with line 66, the authors go back and forth between the specific context of Senegal, and the general context in SSA. I suggest that they move from general to specific.

Please note that both the comments entered here and answers to the questions below constitute the report, bearing your name, that will be forwarded to the authors and published on the site if the article is accepted.

Level of interest - An article whose findings are important to those with closely related research interests

Quality of written English – Acceptable

I declare that I have no competing interests.

Response to reviewers

UNIVERSITY OF CALIFORNIA, LOS ANGELES **UCLA**

BERKELEY - DAVIS - IRVINE - LOS ANGELES - MERCED - RIVERSIDE - SAN DIEGO - SAN FRANCISCO SANTA BARBARA - SANTA CRUZ

SCHOOL OF PUBLIC HEALTH

P.O. BOX 951772
LOS ANGELES, CALIFORNIA 90095-1772

September 30, 2016

Dear BMC Pregnancy and Childbirth Editorial Committee,

We appreciate the thoughtful suggestions from reviewers for improvement of our manuscript entitled "Examining the mechanisms by which women's status and empowerment affect skilled birth attendant use in Senegal: A structural equation modeling approach". We have attempted to address each of the critiques in the attached, revised manuscript and have provided a list of our revisions and responses to reviewers' comments (below).

We believe that the paper addresses important gaps in research and practice, by identifying and exploring the key drivers to improve Skilled Birth Attendant (SBA) use in Sub-Saharan Africa. Women's status and empowerment have been recognized as critical factors to address in the SDG era, not only for the reduction of maternal mortality but also for the broader social and economic development. This paper highlights the importance of these synergetic efforts and recommends interventions for women to have better education, delay marriage and promote progressive gender-role attitudes.

As a recap, the study is the result of a secondary data analysis of Demographic and Health Surveys in Senegal in 2010-11, and the analysis employed a structural equation modeling approach to examine the complex pathways linking women's status, empowerment and SBA use. The results demonstrated the multidimensional composition of women's status and empowerment as they influenced SBA use directly and indirectly.

We are excited to share this important evidence that is meaningful for future research, policy and program interventions, and look forward to receiving your feedback.

Sincerely,

Kyoko Shimamoto, MPH PhD
Maternal, Newborn and Child Health Specialist
UNICEF East Asia and Pacific Regional Office
(Current affiliation)

UCLA School of Public Health
(Former affiliation during this work)

Jessica D. Gipson, MPH
PhD Assistant Professor
UCLA School of Public Health

Response to Reviewer 1 comment:

1. The study is based on a country (Senegal) and may be inappropriate to consider that the study is on West Africa. Either refer the study for "…a country in West Africa" or "in Senegal" in the title.

We revised the title as suggested.

2. A major omission of the paper is a conceptual framework, which may serve as the basis of specifying the path diagram. Without a conceptual framework, it is difficult to follow the construct of empowerment, rationales for specifying causal pathways, and justifying the variables included in the SEM. Discussion on the conceptualization, operationalization and the measurement of women's status and empowerment is also scanty in the paper. The authors clearly recognize the problems of "inconsistent operationalizations and measurements of empowerment" (p. 6, line # 83) in the gender literature. A conceptual framework will fill those voids.

In the Background, we added explanation of the constructs, operationalization and measurement of women's status and empowerment. We also added a conceptual framework in reference to gender literature/theory in the Method section.

3. I do not consider that the inference, "Overall, women's status and empowerment were positively related to SBA use," is appropriate when the result shows decision-making, which is the key component in defining women's status based on actual experience, was not related to SBA use. Among the three construct variables of status and empowerment, the other two are based on perceived attitudes. The interconnections or correlations among the three latent variables are not clear from the presented results. If the two attitudinal latent variables are dropped, does the relationship between decision-making and SBA become significant? Understanding the relationships among these three constructs is critical and relevant for policy implications of this study in advancing women empowerment and gender equity.

We revised the abstract, acknowledging variations across measures of women's status and empowerment. We also revised and clarified the results on the interrelation among the three latent variables based on the present analysis, particularly from the factor analysis and SEM results. Our earlier paper (Shimamoto&Gipson 2015) using regression analysis demonstrated a significant bivariate relationship between decision-making and SBA. However, another manuscript includes the results on hierarchical multivariate regression (not yet published), showing insignificant relationship between decision-making and SBA in a model excluding the two attitudinal measures. Related to the Reviewer 2 comment # 5 too, we revised and highlighted the policy implication related to this somewhat unexpected result in the Discussion section.

4. The study did not explore the linkages between the age at marriage and gender role attitude. Nevertheless, the Discussion section suggested, "This study highlights the important linkages such that older age at first marriage is related to progressive gender-role attitudes, leading to higher likelihood of SBA use and likely better delivery outcomes." I think this beyond the scope of the study results.

In the results section, the linkage between the age at first marriage and gender role attitudes was stated – "Progressive attitudes towards violence were related to <u>older age at first marriage</u>, urban residence,

higher wealth and husband's higher education relative to wife". This result was shown in Table 3, Column 4 – age at first marriage is significantly and positively related to attitudes towards sex negotiation, while insignificant with attitude towards violence or decision-making.

Other suggestions:

1. Label Figure 1 with variable label, not variable name.

We modified the Figure that was developed by Mplus which automatically uses the variable name in the diagram.

2. P.5-6, states, "Examination of the linkages between women's empowerment and SBA use is particularly important in Senegal, where continuous social and development efforts towards gender equality are resulting in gradual shifts in gender norms and relations. For example, women's higher social status is a critical determinant for maternal health care seeking [27, 69 28]." The logical connection between the first sentence and the linking following sentence is not clear to me. Consider the "for example" sentence that is linked to Senegal's context.

Revised the concerned sentence to clarify that the examples/references indeed are from Senegal.

3. May consider changing the text,

   "All the analyses were conducted accounting for individual weights, clusters (i.e., Primary Sampling Unit), and sample strata using the survey analysis commands" (p.13) to:

   "All the analyses were conducted accounting for individual weights and multi-stage stratified cluster survey design for addressing disproportionate (urban-rural) sampling and design-effects >1.00 in the DHS data"

   The use of text "using the survey analysis commands" sound like using the software as a black box.

We revised the concerned sentence in reference to the DHS Sampling and Household Listing Manual (2012).

4. Specifying p<. 01 is sufficient for portraying a meaningful statistical significance level (less than 1%). No need to specify *** p <0.001.

We noted that there is no specific guideline in the BMC in this respect and had followed a latest model article in this journal such as Joshi et al (2014) using DHS data and specified p < 0.001. Also referred to other scholars as a model such as Singh (2015).

Response to Reviewer 2 comment:

- Major Compulsory Revisions

1. The introduction, though well written, can be condensed and tightened. For instance, they state that the literature linking empowerment on health is "remarkably scant", but they already cited several sources in the previous paragraph (beginning with line 54) on associations between women's social status and health outcomes, and then spend the next few paragraphs talking about all of the various studies linking empowerment indicators and care delivery use both in Senegal and elsewhere in SSA. They can better organize the introduction to more succinctly summarize the existing body of

literature on empowerment measures and delivery care use (which should not be referred to as 'scant') before moving to the methodologic limitations with the previous studies that helped to justify the need for the current study.

We condensed the introduction/background as suggested. We also revised the concerned sentence/phase by saying that "the literature is insufficient…to provide a coherent inference" as supported by the proceeding paragraphs outlining evidence gaps, as well as inconsistency in existing evidence.

2. While authors acknowledge the limitation of dropping unmarried women in the limitations section, this is an important limitation that needs to be better addressed. To contextualize this limitation and how it affects the generalizability of their findings, it would be useful to know the proportion of births that occur within and outside of the context of marriage in Senegal.

We agree with the concern and clarified this limitation in the Discussion section. We fully agree that this omission needs to be addressed in programs and data collection efforts, there is a persistent limitation in that many of the empowerment proxy measures are only asked of currently married women. We also noted the proportion/breakdown of births within and outside of marriage/union (7.1% outside of current marriage/union, i.e., 92.9% in marriage/union) in Senegal and that the vast majority of births occurred in marriage/union.

3. The authors could more clearly lay out their hypothesized conceptual framework in the introduction or methods, along with supporting literature.

Per the comment # 2 by the Reviewer 1, we added a conceptual framework in reference to gender literature/theory in the Method section.

4. In methods, "getting permission to go" to the healthcare provider seems like a measure of household decision-making, rather than a control measure of perceived difficulty accessing health care. The authors should better justify the decision to categorize things as they did.

We agree that the two measures have interconnections in concept. We added clarifications in the method section to provide our justification in this analysis. As stated, perceived difficulty in accessing health care asks about four different aspects including one on "getting permission" – which is related to "decision-making" while other questions address other aspects such as financial and geographic access.

5. In the discussion starting at line 289, the authors discuss how the use of SEM "provided the simultaneous comparison of several empowerment dimensions on SBA use, clearly outlining variations in the magnitude and significance of each domain." Yet, the authors do not elaborate at all on the implications of the variations in the magnitude and significance of each domain for policy and programs.

We revised the concerned paragraph, by adding policy and programmatic recommendations, based on the comparative results showing that among the multiple empowerment domains, attitudes towards sex negotiation is the most influential on SBA use in Senegal and this should be a priority.

Other Revisions

1. The authors often alternate between using ‘Africa’ and ‘Sub-Saharan Africa’ (SSA) (for example, final sentence in first paragraph of background). I recommend that they are consistent, use ‘Sub-Saharan Africa’ throughout.

We replaced “Africa” to “sub-Saharan Africa (SSA)” for consistency.

2. The last sentence in background (lines 101-102) is awkward and could be stated more clearly.

We revised the concerned sentence for better clarity.

3. I recommend careful editing throughout. A few examples (which are by no means a comprehensive list) include:
    a. In the fifth paragraph of the background, first sentence: “women” is repeated twice (line 60).
    b. In the last sentence in that paragraph (line 65), punctuation needs to be fixed at the end.
    c. Seventh paragraph of the background starting with line 73), the authors should rewrite with consistent use of tenses.
    d. Results section, descriptive results, final sentence (lines 214-215). Writing is awkward. It may read better to say “…and had on average less than two years of formal education.”

We revised to address the list above and edited other parts of the manuscript, mostly the Background section.

4. The labeling of the latent variable SEM analysis (figure 1) could be improved.

In response to the Reviewer 1 comment too, we modified the Figure that was developed by Mplus which automatically uses the variable name in the diagram. The space is limited and it is unable to have a longer variable name to fit in the circle.

5. Line 225---there is a reference to figure 2, which I assume is meant to be for figure 1.

The numbering of the figures was updated per the inclusion of additional figure.

6. In the Background, starting with line 66, the authors go back and forth between the specific context of Senegal, and the general context in SSA. I suggest that they move from general to specific.

The concerned paragraph was summarized and combined with the preceding one, of which we reorganized the flow from general to specifically Senegal.

Reviewer reports – 2nd round

Reviewer 1: Sheri Weiser

The authors have successfully addressed my original concerns. There are a few minor revisions recommended as outlined below.

1) In abstract, first sentence of results is incomplete, ending with “while,”

2) In the introduction overall, much of the literature review focuses on maternal health care use more broadly but the study question is on SBA use. If there is not specific literature on SBA use, the authors should better clarify this gap in the literature. If there is specific literature, they should clarify this in their literature review. For example, in the paragraph including line 156—I am not clear on whether authors are referring to “maternal health care use” more broadly, or to use of SBA? Throughout the rest of that same revised paragraph, it would be good to clarify which specific health care use outcomes were addressed in each study, and whether there is specific literature on use of SBA? In the last few sentences, the term “delivery care” can also be better specified. The next paragraph then goes into the gaps in the literature on SBA use, but SBA use was not mentioned at all in the previous paragraph.

Reviewer 2: Saifuddin Ahmed

The authors satisfactorily address the reviewers’ comments and suggestions. The study will advance our knowledge base in understanding the multidimensional construct of women’s empowerment and its influence on uptaking maternity care in a low-income country setting in SSA. This is a well-structured manuscript.

I have two minor suggestions:

The paper contributed the impact of education, subtly in biased tone in some places, “through women’s empowerment and gender equity” promotion. Education also may contribute through familial endowment and knowledge advancement in maternal health care needs and life threatening complications.

Remove “, while.” from p.2 line 17.

Response to reviewers – 2nd round

UNIVERSITY OF CALIFORNIA, LOS ANGELES **UCLA**

BERKELEY - DAVIS - IRVINE - LOS ANGELES - MERCED - RIVERSIDE - SAN DIEGO - SAN FRANCISCO SANTA BARBARA - SANTA CRUZ

SCHOOL OF PUBLIC HEALTH

P.O. BOX 951772
LOS ANGELES, CALIFORNIA 90095-1772

December 8, 2016

Dear BMC Pregnancy and Childbirth Editorial Committee,

We appreciate the thoughtful suggestions from reviewers for further improvement of our manuscript entitled "Examining the mechanisms by which women's status and empowerment affect skilled birth attendant use in Senegal: A Structural Equation Modeling approach". We have attempted to address their feedback in the attached, revised manuscript. We have also provided a list of our revisions and responses to reviewers' comments (below).

We are excited to share this important evidence that is meaningful for future research, policy and program interventions, and look forward to receiving your feedback.

Sincerely,

Kyoko Shimamoto, MPH PhD
Maternal, Newborn and Child Health Specialist
UNICEF East Asia and Pacific Regional Office
(Current affiliation)
UCLA School of Public Health
(Former affiliation during this work)

Jessica D. Gipson, MPH PhD
Assistant Professor
UCLA School of Public Health

Response to Reviewer 1 comment:

1. In abstract, first sentence of results is incomplete, ending with "while,"

We corrected the sentence to be complete, by removing the "while" (per Reviewer 2 comment too).

2. In the introduction overall, much of the literature review focuses on maternal health care use more broadly but the study question is on SBA use. If there is not specific literature on SBA use, the authors should better clarify this gap in the literature. If there is specific literature, they should clarify this in their literature review. For example, in the paragraph including line 156—I am not clear on whether authors are referring to "maternal health care use" more broadly, or to use of SBA? Throughout the rest of that same revised paragraph, it would be good to clarify which specific health care use outcomes were addressed in each study, and whether there is specific literature on use of SBA? In the last few sentences, the term "delivery care" can also be better specified. The next paragraph then goes into the gaps in the literature on SBA use, but SBA use was not mentioned at all in the previous paragraph.

We clarified and revised the Introduction, as suggested above. In our literature review, we included papers that examined not only SBA use but also "institutional delivery" and also "delivery care use at health facility". These two measures are generally understood and examined as indicators for "delivery care use" in the DHS and other major surveys. These two indicators overlap substantially, especially in low-resource settings such as Senegal, hence we considered the both measures as our outcome of interest in our literature review. Thus we replaced the phrase of "maternal health care use" with "delivery care use" to present evidence with respect to both of these measures.

Response to Reviewer 2 comment:

1. The paper contributed the impact of education, subtly in biased tone in some places, "through women's empowerment and gender equity" promotion. Education also may contribute through familial endowment and knowledge advancement in maternal health care needs and life threatening complications.

We clarified and revised sentences in the Discussion section concerning the direct effect of education on SBA use.

2. Remove ", while." from p.2 line 17.

Per the comment #1 by Reviewer 1, we revised the concerned sentence.
